# Dentists’ Benevolent Association

**Published:** 1884-10

**Authors:** A. D. Dudley


### Dentist's Benevolent Association.

The first annual meeting of the above orginization was held at Sweet Springs, Mo., July 10th, 1884, Dr. C. H. Darby in the Chair. About twenty-five members were in attendance. Annual report of Secretary and Treasurer received and approved. The following officers elected for ensuing year:

President: Dr. C. H. Darby, St. Joseph, Mo.

Vice-Presidents: Dr. F. Swap, Booneville, Mo.; Dr. J. J. R. Patrick, Belleville, Ill.; Dr. W. H. Eames, St. Louis, Mo.; Dr. R. E. Nickles, Salina, Kan.; Dr. J. W. Reed, Butte City, Montana T.; Mr. F. X. Combs, Chicago, Ill.

Secretary and Treasurer: Dr. R. I. Pearson, Kansas City, Mo.

Board of Directors: Dr. A. H. Thompson, Topeka, Kan.; Dr. J. A. Price, Weston, Mo.; Dr. J. M. Austin, St. Joseph, Mo.; Dr. Geo. H. Cushing, Chicago, Ill.; Drs. G. W. Tindall, C. B. Hewit, J. D. Patterson, H. S. Thompson, A. C. Schell, J. S. Letord, Kansas City, Mo.

This Association is now in practical working order, and is duly authorized by charter from the State of Missouri to carry out the objects of its orginazation. Composed exclusively of persons identified with the dental profession, and having no salaries or other excessive expenses to meet, it offers to those eligible to its benefits a cheap and reliable means of providing for their families in case of death. For further information address R. I. Pearson, Secretary, 2206 Troost Ave., Kansas City, Mo.

The Association, the proceedlngs of which are given above, is one of special importance to the profession and is worthy their earnest attention and consideration. If carried out according to its intent, and it doubtless will be under its present organization, it would be productive of much good and especially so if encouraged and sustained by the best part of the profession. We have no hesitancy in recommending it to the profession. It will certainly enable its members to avoid serious financial embarrassment that has occasionally fallen on the families of some very worthy deceased members of the profession. We shall have occasion to refer to this subject again and for the present only ask that it receive kindly consideration.

The annual meeting of the New England Dental Society will be held in Boston, Thursday and Friday, October 2nd and 3rd. An interesting programme of practical subjects has been prepared and the officers earnestly invite every member to be present at the meeting—to be present from the beginning to the end. They suggest that every member place the appointment on his engagement book. Programmes will be forwarded to those who desire them. A. D. Dudley.

